# A Game Model and Fault Recovery Algorithm for SDN Multi-Domain

**DOI:** 10.3390/s25010164

**Published:** 2024-12-30

**Authors:** Tao Xu, Chen Chen, Kaiming Hu, Yi Zhuang

**Affiliations:** 1 The College of Computer Science and Technology, Nanjing University of Aeronautics and Astronautics, Nanjing 211106, China; xutao0920@163.com (T.X.); cyz2020@nuaa.edu.cn (C.C.); kaiming-hu@nuaa.edu.cn (K.H.); 2 Jiangsu Automation Research Institute, Lianyungang 222061, China

**Keywords:** fault recovery, software-defined network (SDN), game domain

## Abstract

Software-defined networking (SDN) offers an effective solution for flexible management of Wireless Sensor Networks (WSNs) by separating control logic from sensor nodes. This paper tackles the challenge of timely recovery from SDN controller failures and proposes a game theoretic model for multi-domain controllers. A game-enhanced autonomous fault recovery algorithm for SDN controllers is proposed, which boasts fast fault recovery and low migration costs. Taking into account the remaining capacity of controllers and the transition relationships between devices, the target controller is first selected to establish a controller game domain. The issue of mapping the out-of-control switches within the controller game domain to the target controller is transformed into a linear programming problem for solution. A multi-population particle swarm optimization algorithm with repulsive interaction is employed to iteratively evolve the optimal mapping between controllers and switches. Finally, migration tasks are executed based on the optimal mapping results, and the role transition of the target controller is completed. Comparative experimental results demonstrate that, compared to existing SDN controller fault recovery algorithms, the proposed algorithm can balance the migration cost of switches and the load pressure on controllers while reducing propagation delay in SDN controllers, significantly decreasing the fault recovery time.

## 1. Introduction

With the rapid development of the Internet of Things (IoT), the Wireless Sensor Network (WSN) [1] is becoming increasingly important. As WSN scales up, optimizing the flexibility, reliability, and energy consumption of the overall network becomes critical [2]. SDN [3], as an emerging approach for WSN, is increasingly favored by researchers in data centers and network architecture due to its ability to address complex issues more quickly and flexibly through the separation of control and forwarding mechanisms, as well as its philosophy of programming network functions. The abstract and centralized control plane can effectively enhance the efficiency of network resource allocation, while the programmable forwarding plane achieves software–hardware separation, making the entire WSN architecture more flexible and scalable. However, with the diversification of the network deployment environment, the influence of a complex environment and the expansion of network scale pose numerous challenges to the control plane composed of a single controller. To ensure high network availability, how to guarantee the high reliability of SDN controllers has become one of the research focuses.

The software-defined approach divides WSNs into a centralized control plane and a programmable one, which offers more flexible network management. Controllers, as the fundamental components of the control plane that manage the data plane, have a significant impact on the overall dependability of the network due to their inherent reliability. Therefore, sensor network reliability issues within the SDN architecture can be categorized into two types: those pertaining to the control plane, influenced by controllers, and those associated with the data plane, encompassing forwarding devices and links. The control layer is logically centralized, and when controllers lose control over the data plane, it can result in localized network failures at best and potentially cause the entire network to collapse in severe cases. One research approach for the reliability of the control plane is to establish a controller cluster. Distributed controllers provide redundant backups of state information, and the utilization of distributed data storage technology can mitigate network malfunctions caused by individual controller failures, thereby enhancing control plane reliability. For instance, ONOS [4] employs a distributed control plane where state information distributed across multiple controllers is managed and maintained for consistency by Zookeeper [5]. Another approach involves setting up a dedicated set of servers to store the state information of the controller, and the controller only needs to read the state information from these servers to carry out fault analysis [6].

Although the network based on SDN architecture has resolved the problems of overcrowding and closure of WSN, the hierarchical network itself has encountered challenges in automatic management and reliable operation. A clustered control plane enhances the network’s fault tolerance against single points of failure in controllers, making the network more stable and reliable when facing controller failures, but there are still some unresolved issues. Challenges remain in aspects such as collaboration and synchronization between controllers, as well as rapid fault detection and autonomous recovery. These problems become even more prominent in large-scale and complex WSN environments. Therefore, it is necessary to research an effective fault monitoring and autonomous recovery mechanism for multi-domain controllers in SDN to improve the reliability of the WSN system.

The main contributions of this paper are as follows: (1) In order to solve the switch migration problem caused by unrecoverable failure of SDN controller, a solution based on game-enhanced model is proposed, and a game model for SDN multi-domain controller is constructed to provide a theoretical basis for the remapping between switches and controllers; (2) A method for constructing a controller game domain is proposed: the target controller and out-of-control switch are selected based on the remaining capacity of controller and the transition relationship between devices to establish a controller game domain; (3) The issue of mapping between the out-of-control switch and the target controller in the controller game domain is transformed into a linear programming problem for solution, and the migration cost of the out-of-control switch and the load pressure on the controller are comprehensively considered. A multi-population particle swarm optimization algorithm with repulsive interaction [7] is employed to determine the optimal mapping between controllers and switches; (4) Aiming at the potential unrecoverable faults that may occur in SDN controllers, an SDN controller fault Multi-Autonomous Recovery Algorithm (SDN-MARA) based on multi-domain controller game model is proposed, which boasts high fault detection accuracy and rapid recovery. Finally, comparative experiments demonstrate that the proposed model and algorithm in this paper can balance the migration cost of switches and the load pressure on controllers while reducing propagation delay in SDN controllers, significantly improving fault recovery efficiency.

## 2. Related Work

The control plane is the most vulnerable component of the entire network, and the layout of controllers affects aspects such as network delay, energy efficiency, load, and network resilience, which has garnered significant attention from researchers. Currently, research on the reliability technology of the control plane in SDN architectures primarily focuses on controller deployment, fault detection, and fault recovery.

Scholars have modeled and analyzed the Controller Placement Problem (CPP) in SDN based on graph theory, game theory, queuing theory, etc. For example, Ma et al. [8] suggested that, based on the meta-heuristic algorithm, the discrete African Vulture optimization algorithm (AVOA) was solved by low-complexity multi-point and two-point permutation methods in the search stage and the development stage to optimize the controller placement scheme. Mohanty S, et al. [9] proposed to prevent the significant increase in worst-case delay and disconnection by predicting failures so as to minimize the worst-case delay between the switch and its backup controller, as well as between controllers.

Current research on the CPP mainly involves three objectives: minimizing network delay and controller deployment costs and maximizing network reliability. Ma proposed an improved K-means algorithm based on an artificial bee colony (ABCK) [10] to efficiently calculate the optimal or near-optimal solutions. To minimize propagation delays between controllers and switches and maintain load balance between controllers. Thalapala V S et al. put forward a highly reliable, delay-optimized controller placement (HR-DO) algorithm [11], which can be used to optimize the number of controllers required in a network and reduce network delay. It also improves the reliability and robustness of SDN while minimizing the delay in the worst-case scenario. Mycek M et al. raised an optimization model [12] to jointly upgrade the placement of primary and standby controllers in long-distance SDN networks, which can enhance the elasticity and dependability of the network against node attacks. Xu H proposed a multi-controller placement strategy based on the improved Harris Hawks optimization algorithm [13]. Considering the total network delay, node reliability, link failure rate, and total placement cost, the switching mode of dynamic adaptive global search and local search can enhance the diversity of CPP schemes and improve placement reliability.

Network deployment cost and energy consumption are also important factors affecting control plane reliability. Solutions to minimize controller deployment costs can be summarized as static and dynamic methods. Static methods take into account the optimal number, type, location of controllers, and interconnection information between switches and controllers. Maity et al. considered the control plane and IoT traffic and proposed an energy-saving controller placement scheme, EnPlace [14]. Compared to existing solutions, EnPlace can significantly save energy. Maaloul et al. focused on studying energy-efficient routing mechanisms for traffic in SDN-based operator Ethernet networks. A heuristic algorithm [15] is put forward to select the switches and links to be disabled according to predefined criteria. Taking the constraint of link capacity into consideration, existing traffic is rerouted through the best available path.

Relevant scholars have studied the fault recovery of controllers in SDN distributed architectures from controller placement and migration of out-of-control switches. One research focus is on controller deployment and optimization, where the network is abstracted as an undirected topology graph, and the controller deployment can be regarded as a partition mapping problem for the topology. Under the existing network topology, controllers need to comprehensively consider the impact of inter-device hops and propagation delays. The selection of controller locations is determined by the positions of switch nodes. The general solution is based on technologies such as k-means [16] and clustering [17]. The disadvantage of this method is that it overly idealizes the network without fully considering the impact of the network performance index. When considering network metrics such as communication delay, throughput, and load, one solution is to use heuristic methods [18] or incorporate machine learning mechanisms to improve population optimization algorithms [19], seeking the optimal deployment strategy for controllers. Another research focus is on methods based on the migration of out-of-control switches [20], where a control plane composed of multiple controllers supports the migration of switches within the controller fault domain. Therefore, designing a migration mechanism is the primary consideration to ensure rapid recovery of network functions. Migration algorithms conditioned on controller load are currently a research hotspot [21]. Dynamic migration mechanisms can be dynamically adjusted according to network operation to adapt to the large, complex network environment. No matter which fault recovery method has its own specific applicable environment and network conditions. During fault recovery, factors such as algorithm efficiency, switch migration cost, and delay need to be comprehensively considered.

In summary, SDN, as a programmable, centralized, and scalable WSN architecture with flexible scheduling, has unique technical advantages in dealing with challenges of network reliability. However, due to the continuous expansion of the network scale, the types of possible faults are also increasing. Based on traditional WSN mode, most of the existing research focuses on link-level fault detection and recovery technology and lacks research results that can make good use of the characteristics of SDN layered architecture. Not only do they have certain limitations in application scenarios, but there is also the problem of controllers in SDN existing faults that are difficult to recover timely. On the other hand, the existing research work has the problem of insufficient speed and mapping balance between fast switches and controllers. Therefore, the network based on SDN layered architecture still faces challenges in automated control as well as highly reliable operation.

## 3. Reliability Game-Enhanced Model for SDN Multi-Domain Controllers

To overcome the potential challenges in timely fault recovery and high-reliability operation of SDN controllers, this paper constructs a game enhancement model for SDN multi-domain controllers, providing a foundation for resolving issues related to fault detection and autonomous recovery of SDN controllers.

The control plane is logically divided into multiple control domains, with each domain consisting of a local controller and several switches. The local controller is responsible only for managing the switches within its own domain, while different control domains interact and synchronize information through east–west interfaces between local controllers. If a local controller fails, the switches within its domain will lose control. Therefore, it is necessary to remap the switches in the failed controller’s domain to other functioning controllers to ensure stable connectivity and high availability of the network system.

To achieve the remapping between controllers and switches, the first step is to select a node that is either within the same control domain as the failed controller or in an adjacent control domain, ensuring that this node has sufficient remaining capacity. This node will serve as the new target controller, responsible for managing the control and data flows of the switches that were previously managed by the failed controller. To facilitate effective remapping, it is necessary to establish a controller game model [22]. The game domain refers to the analytical unit comprising participants and the combinations of actions they select over a specified timeframe. In the game domain, players can be either individuals or organizations, and they make decisions based on their own interests and goals. The collective actions chosen by all players during a concurrent period are defined as an action combination that reflects the decision-making outcomes of the players. The controller game domain is a temporary network decision domain that is autonomously formed as needed. When a controller failure occurs in the network, the switches within this domain will autonomously select a new controller to manage their data and control flows.

The SDN-oriented controller game domain consists of a collection of out-of-control switches and controllers participating in the decision-making process. When a controller fails, the functioning controllers within the domain or adjacent domain will take over the control and management of the switches belonging to the failed controller. They will collaborate with other neighboring controllers to negotiate and determine the optimal controller mapping scheme.

**Definition** **1.** *The SDN multi-domain controller network is represented as an undirected graph G, as shown in Equation (1).*

(1) $$G=\left(V,E,C,Q\right)$$

*where V represents the set of switches in the network.*$V=\left\{v_{1},v_{2},\ldots ,v_{n}\right\}$*, n = |V| is the number of switches; E denotes the set of data links in the network; and C shows the set of controllers.* $C=\left\{c_{1},c_{2},\ldots ,c_{m}\right\}$*, m = |C| is the number of controllers; Q indicates the set of control domains.*$Q=\left\{{\langle c_{1}^{i},v_{c_{1}^{i}}^{j}\rangle }_{1},{\langle c_{2}^{i},v_{c_{2}^{i}}^{j}\rangle }_{2},\ldots ,{\langle c_{q}^{i},v_{c_{q}^{i}}^{j}\rangle }_{q}\right\}$*, q signifies the number of control domains.*${\langle c_{k}^{i},v_{c_{k}^{i}}^{j}\rangle }_{k}$*denotes that the k-th control domain consists of the mapping from the controller set* $c_{k}^{i}$ *to the switch set* $v_{c_{k}^{i}}^{j}$*, where k represents the current domain number and j indicates the number of switches managed by the controller indexed by i within the current control domain. To fully leverage the characteristics and performance of switches and controllers in SDN, while also considering the requirements for load balancing and fault tolerance within the network, a hybrid mapping approach is adopted between switches and controllers.*

The constructed game model is based on the following assumptions: (1) each controller has the same processing capability, and there is no variability between requests from different switches; (2) during normal operation, the local controller makes independent decisions. When a controller fails, the out-of-control switches within its domain become bargaining chips in the game, prompting the selection of a target controller with sufficient remaining capacity to join the game. Participants in the game must also consider the propagation delay of the controllers and the cost of migration to maximize their respective interests.

**Definition** **2.** *The mapping matrix* $M_{i,j}$ *represents the mapping relationship between switch*j*and controller*i*within the control domain, as shown in Equation (2).*

(2) $$M_{i,j}=\left\{\begin{array}{c} 1,\;v_{j}\;is\;mapped\;to\;c_{i}\;\\ \;0,\;v_{j}\;is\;not\;mapped\;to\;c_{i} \end{array}\right.$$

To ensure the accurate execution of SDN controller routing strategies, it is necessary for controllers to exchange their port status tables and mapping matrices. The switching strategy designed in this paper are as follows: (1) switch the routing table during network initialization to achieve global consistency; (2) send global update information when routing information in the control domain changes; and (3) in case of packet loss, set the timeout resend strategy. If no confirmation message is received within the set time, the port state table and mapping matrix are retransmitted.

**Definition** **3.** *The remaining capacity* $R_{i}$ *of a controller is defined as the difference between the expected maximum reception rate of the controller within the control domain and the actual request rate of the switches. The calculation method for the remaining capacity $R_{i}$* *of each controller is shown in Equation (3).*

(3) $$R_{i}=\epsilon_{i}-\sum_{j}\partial_{j}\cdot M_{i,j}$$

*where $\epsilon_{i}$* *represents the expected maximum reception rate of controller i, and $\partial_{j}$ denotes the actual request rate of switch j.*

**Definition** **4.** *Controller game domain (CGD) is a decision-making unit that the system autonomously constructs when a controller in the network fails to determine the migration of out-of-control switches through normal controller game. CGD in SDN consists of a set of out-of-control switches and neighboring normal controllers involved in decision-making. The controller game domain is denoted as Equation (4).*

(4) $$CGD=\left\{c_{k}^{i},{\;v}_{c_{k}^{i}}^{j}\right\},\;i=\left(1\sim m\right),\;j=\left(1\sim n\right),\;k=\left(1\sim q\right)$$

**Figure 1:**
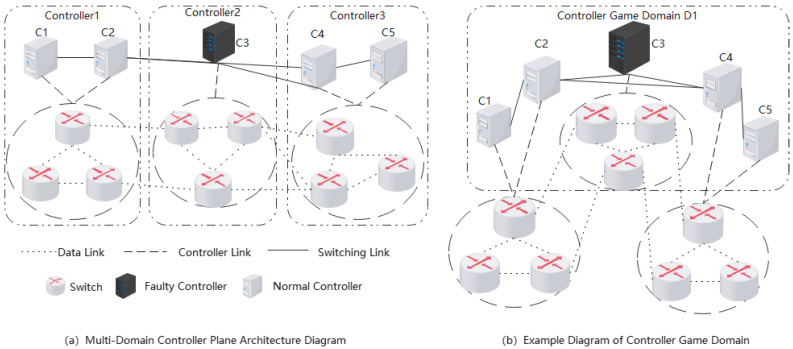
Multi-domain controller architecture diagram.

For example, as shown in Figure 1a, in a multi-domain controller system with five SDN controllers and multiple switches forming three control domains, if controller $c_{3}$ fails at a certain moment, as illustrated in Figure 1b, the switches within control domain $q_{2}$ will become out of control. At this point, the neighboring controllers of $c_{3}$ and the out-of-control switches will together form the controller game domain ${CGD}_{1}$, shown as Equation (5).

(5) $${CGD}_{1}=\left\{c_{1}^{i},\;{\;c}_{3}^{i},\;v_{c_{1}^{i}}^{j},{\;v}_{c_{3}^{i}}^{j}\right\}$$

In SDN, the initiation of game activities and the formation of game domains are prompted by the detection of a failed controller. This controller can be an effective one within the same domain or a neighboring one, referred to as the game-initiating controller. Each controller is assumed to participate in only one game activity at a time to ensure isolation between different controller game domains.

If there is only a single controller within the control domain and it fails, it will directly notify the neighboring ones to perform fault detection and recovery. When there are two or more controllers in the control domain, if one fails, others can quickly detect it. The out-of-control switches within the domain will then be taken over by the functioning controllers, which may lead to excessive load on those controllers. Therefore, it is necessary to appropriately reduce the number of switches assigned to them, marking some of them as out-of-control and notifying neighboring controllers to establish a game domain, thus entering the fault recovery phase. This situation is similar to handling the failure of a single-controller control domain. Therefore, this paper focuses on the activities within a single-controller game domain.

**Definition** **5.** *The switch migration cost* $P_{t}$ *represents the total cost associated with the migration of an out-of-control switch from the selected target controller to the establishment of a new reliable channel. This cost specifically includes two components: the channel creation cost $P_{s}$* *and the data migration cost $P_{f}$:*

(1) $P_{s}$: When a switch transitions to a new controller, it needs to establish a control channel with the new controller. This process includes sending a request to establish a connection with the new controller and reporting the switch’s state information, such as flow tables and port states, to the new controller. Once the request is accepted, the switch receives new flow table rules or maintains existing rules and continues to forward data traffic. Thus, the cost of creating the new channel $P_{s}$ is calculated in two phases: In the first phase, after determining the switch $v_{j}$ to be migrated, $v_{j}$ needs to send a connection establishment request to the target migration controller $c_{i}$ through the game-initiating controller. In the second phase, after $c_{i}$ accepts the request, the cost of information exchange during the process of directly establishing the channel between $v_{j}$ and $c_{i}$ includes the cost of message authentication and the overhead of establishing the channel between the controller and the switch. The calculation method is shown in Equation (6).

(6) $$P_{s}=\left(\tau_{q}\cdot Tq+\tau_{c}\right)\cdot \frac{{RTT}_{i,j}}{h\left(i,j\right)}$$

In the first phase, $\tau_{q}$ represents the packet size of the request sent by the game-initiating controller, and Tq is the number of times that $v_{j}$ sends a connection establishment request. In order to ensure the authentication process of secure communication, the authentication method based on TLS security protocol [23] is adopted to verify the identity of the controller and switch involved. In the second phase, $\tau_{c}$ denotes the average packet size during the establishment of the channel between the controller and the switch, ${RTT}_{i,j}$ is the round-trip time delay from controller i to switch j, and $h\left(i,j\right)$ represents the hop count, indicating the number of hops a packet takes from controller i to switch j within the current game domain.

(2) $P_{f}$: The data migration cost occurs after $v_{j}$ establishes a new channel with $c_{i}$. The controller $c_{i}$ needs to send the flow rules within the domain to the switch $v_{j}$. The calculation method for the migration cost $P_{f}$ is shown in Equation (7), where $\tau_{rule}$ represents the average size of the flow_mod packet.

(7) $$P_{f}=\tau_{rule}\cdot h\left(i,j\right)\cdot M_{i,j}$$

Therefore, the calculation method for the switch migration cost $P_{t}$ is given in Equation (8), where $a_{s}$ and $a_{f}$ are the weights corresponding to the channel establishment cost and the migration cost, respectively.

(8) $$P_{t}=a_{s}*P_{s}+a_{f}*P_{f}$$

In this paper, the framework of a non-cooperative game is adopted when constructing game models and designing algorithms. Each controller is treated as a player with the goal of optimizing the mapping between switches and controllers throughout the game to maximize their respective utility functions. The game here involves incomplete information, that is, each controller and switch have asymmetrical state information of other nodes. Each controller can only obtain the state of its own managed nodes but cannot fully understand the state of others.

**Definition** **6.** *The game for SDN multi-domain controllers is a non-cooperative one with incomplete information, which is defined as a quadruple, as shown in Equation (9).*

(9) $$\Upsilon_{GEA}=\left(C,\left\{I_{i}\right\},\left\{X_{i}\right\},\left\{U_{i}\right\}\right)$$

$C=\left\{c_{1},\ldots ,c_{m}\right\}$ represents the set of players, and each player $c_{i}$ refers to the alternative target controller participating in the game, whose remaining capacity is greater than the threshold selected from the set of failed neighborhood controllers.

$I_{i}=\left\{I_{i1},\ldots ,I_{in}\right\}$ is the cost set for player $c_{i}$. Each cost $I_{ij}$ refers to the price paid by the controller $c_{i}$ to accept the switch $v_{j}$ migration request through the security protocol, and the calculation method is shown as Equation (10). It is mainly composed of the switch migration cost $P_{t}$ and the load pressure $\Omega_{i}$ generated by the target controller due to the switch migration. Switch migration is performed using TLS security protocol, including the process of re-establishment of connections in the network and routing table issuance, and the security and integrity of the network are maintained through keys. The calculation method for $\Omega_{i}$ is shown in Equation (11):

(10) $$I_{ij}=\left\{\begin{array}{c} P_{t}+\Omega_{i},\;x_{i}\left(I_{ij}\right)=1\\ 0,\;x_{i}\left(I_{ij}\right)=0\; \end{array}\right.$$

(11) $$\Omega_{i}=\delta_{v_{j}}\cdot pre\left(v_{j}\right)$$

where $pre\left(v_{j}\right)$ represents the expected request rate of switch $v_{j}$, and $\delta_{v_{j}}$ denotes the expected average packet size.

$X_{i}=\{x_{i}\left(I_{i1}\right),\ldots ,x_{i}(I_{in})\}$ is the strategy set of the player $c_{i}$, and each strategy $x_{i}\left(I_{ij}\right)$ determines the actions of the player $c_{i}$ under current cost $I_{ij}$. If $x_{i}\left(I_{ij}\right)=1$ means that controller $c_{i}$ accepts the migration request from switch $v_{j}$, if $x_{i}\left(I_{ij}\right)=0$ means that it rejects it.

$U_{i}$ is the revenue function of the player $c_{i}$, indicating the inverse sum of the costs of a newly connected switch for the same controller. The calculation method is shown in Equation (12):

(12) $$U_{i}(x_{i},X_{-i})=\left\{\begin{array}{c} \sum_{v_{j}\in v_{c_{i}}}{-O}_{j},\;if\;x_{i}\left(I_{ij}\right)=1\;and\;arg\underset{r}{\min}O_{j}=i\\ 0,\;else \end{array}\right.\;s.t.\sum_{c_{i}\in c_{v_{j}}}x_{i}\left(I_{ij}\right)=1$$

where $X_{-i}=X_{1}\times \ldots \times X_{i-1}\times X_{i+1}\times \ldots \times X_{m}$ is the set of strategies of all players except the $c_{i}$. Switch $v_{j}$ can select multiple controllers to make migration game decisions, and different decision costs are represented by $O_{j}$, and then make global decisions according to decision costs to select appropriate controllers for migration. The calculation method of $O_{j}$ is shown in Equation (13).

(13) Oj=atPt+arΩrs.t ∀r,∀k,Dr∩Dk=∅

where $a_{t}$ and $a_{r}$ represent weight factors. Since only a single game domain activity is considered in this paper, for two different controller game domains $D_{r}$ And $D_{k}$, the constraint condition indicates that there is no controller intersection between different game domains.

The game for SDN multi-domain controllers adopts Nash equilibrium strategy to solve the problem of resource allocation and coordination among controllers.

**Definition** **7.** *For any controller* $c_{i}$ *and* $x_{i}^{\prime }\in X_{i}$ *, if strategy* $X^{*}=(X_{1}^{*},\ldots ,X_{m}^{*})$ *satisfies* $U_{i}\left(x_{i},X_{-i}^{*}\right)\geq U_{i}\left(x_{i}^{\prime },X_{-i}^{*}\right)$ *, then* $X^{*}$ *is called Nash equilibrium.*

## 4. SDN-MARA Algorithm

### 4.1. SDN-MARA Algorithm Framework

**Figure 2:**
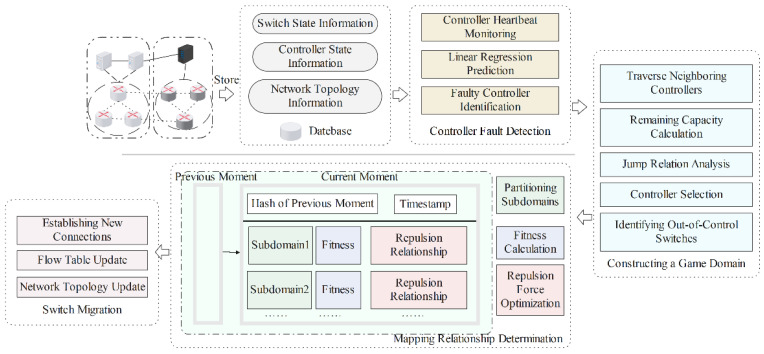
Framework diagram of SDN multi-domain controller fault recovery algorithm.

Based on the aforementioned SDN multi-domain controller game model and its analysis, this paper designs the overall framework of SDN-MARA based on multi-domain controller game model, as depicted in Figure 2. The algorithm comprises fault detection, game domain construction, controller–switch mapping, and switch migration. In the first stage, the faulty controller is detected. In the second stage, when the faulty controller and the out-of-control switch are identified, a game domain is actively constructed, game calculations are performed, and a target controller is selected. In the third stage, the new mapping relationship between the switch and the target controller is established. In the fourth stage, switch migration and target controller role transition are executed to achieve fault recovery.

As shown in Figure 2, multiple SDN controllers and their respective managed switches collectively form a multi-domain SDN network. For a normally operating network, a heartbeat monitoring mechanism is employed to monitor the status of switches and controllers, collecting and analyzing crucial network and device state information, such as heartbeat delay and bandwidth utilization. Through the analysis and prediction of current state information, it detects the presence of faulty controllers. If a faulty controller is detected at a certain moment, a game domain will be constructed for the out-of-control switch.

During the construction of the game domain, it is necessary to traverse the faulty controller’s domain and its neighboring ones. Throughout this traversal, the remaining capacity of each controller is calculated, and the transition relationship is analyzed. If the remaining capacity of a target controller is greater than the minimum expected request rate of the out-of-control switch and the controller has not joined any other game domains, it is added to the set of target controllers; otherwise, the traversal continues with the remaining controllers. The candidate target controllers are then sorted in descending order based on their remaining capacity. Subsequently, all out-of-control switches within the faulty controller’s domain are obtained and joined with the candidate target controllers in the same game activity, completing the construction of the game domain.

In order to determine the mapping relationship, this paper designs a game-enhanced dynamic mapping algorithm that employs a multi-population particle swarm optimization (PSO) algorithm [7] with repulsive interaction to determine the mapping between out-of-control switches and target controllers. In this paper, target controllers are abstracted as particles, and the set of target controllers in the game domain is further divided into several decision subdomains, which helps to break down the original problem into sub-problems, thereby simplifying the solving process. Combined with the repulsive relationship optimization technique, the algorithm records the fitness value at every moment to determine the optimal fitness and enhance the global optimization capability. By applying different inter-swarm interaction factors at different steps, the multi-population particle swarm algorithm with a repulsive relationship can gradually approach the optimal mapping.

The fourth phase of the algorithm is to use the fault autonomous recovery algorithm designed in this paper to achieve fault recovery, including executing switch migration and target controller role transition. Firstly, new connections need to be established. Then, the local controller updates the switch information within its domain and performs flow table update operations. Through these steps, the system autonomously completes the recovery from network faults, allowing the network to reenter a normal operating state.

### 4.2. Optimal Mapping Between Controllers and Switches

When seeking the target controller, it is necessary to consider the balance between the migration cost of out-of-control switches and the propagation delay within the game domain. In this paper, the mapping selection between out-of-control switches and target controllers within the controller’s game domain is transformed into a linear programming problem for solution. Factors such as the migration cost of out-of-control switches and the load pressure on controllers are comprehensively considered in mapping selection. This paper employs a multi-population particle swarm algorithm with repulsive interaction to solve this linear programming problem, aiming to find an optimal mapping scheme. The designed optimal mapping algorithm between controllers and out-of-control switches not only minimizes the migration cost of switches but also ensures that the network load pressure remains within an acceptable range. Based on the game-enhanced model proposed in this paper, the fitness function is calculated according to Equation (10).

In order to achieve a more balanced controller load and shorter task processing delays in the reestablished mapping, it is necessary to design a mapping strategy that takes into account both global and local performance. Since a hybrid mapping strategy can achieve global load balancing and avoid issues such as locally optimal performance, this paper adopts a hybrid mapping strategy.

The repulsive forces between different decision subdomains are divided into repulsive forces of positive, negative and equal. Let the current average fitness value be denoted as $f_{avg}$, and the historical best fitness value as $f_{gb}$. This paper proposes the following three types of strategies X: (1) During the controller game process, positive repulsive force indicates that the switch has a low fitness with the current game domain, and other game domains should be prioritized. That is, when under positive repulsive force, both $f_{avg}$ and $f_{gb}$ of the current decision subdomain are weaker than those of other decision subdomains. The optimization behavior of this subdomain is influenced by other stronger ones and tends to align with their exploration behavior. (2) Negative repulsive force indicates that the switch has a high fitness with the current game domain which should be prioritized. That is, when under negative repulsive force, both $f_{avg}$ and $f_{gb}$ of the current decision subdomain are stronger than those of other subdomains. This subdomain has a strong independent optimization capability and can influence the exploration behavior of other others. (3) Equal repulsive force indicates different results for different judgment conditions. That is, when under equal repulsive force, $f_{avg}$ or $f_{gb}$ of the current decision subdomain is weaker than those of others, while the other is stronger. In this case, the decision subdomains will maintain their respective search areas, thereby keeping both in relatively independent search regions.

The three types of repulsive forces can be evaluated based on two indicator parameters: the current average fitness value $f_{avg}$ and the historical best fitness value $f_{gb}$ of particles in the decision subdomain. Therefore, after introducing the repulsive relationship, for decision subdomains A and B, the calculation methods for particle velocity ${sp}_{A}$ and position ${pos}_{A}$ update are as shown in Equations (14) and (15):

(14) $$\begin{array}{c} {sp}_{A}\left(t+1\right)=w\times {sp}_{A}\left(t\right)+\vartheta_{1}\times r_{1}\times \left(f_{Apb}\left(t\right)-{pos}_{A}\left(t\right)\right)\\ +\vartheta_{2}\times r_{2}\times \left(f_{Agb}\left(t\right)-{pos}_{A}\left(t\right)\right)\;\\ -\frac{\vartheta_{3}}{\left(f_{Bgb}\left(t\right)-{pos}_{A}\left(t\right)\right)}\times natural\left(\;\right) \end{array}$$

(15) $${pos}_{A}\left(t+1\right)={pos}_{A}\left(t\right)+{sp}_{A}\left(t+1\right)$$

${sp}_{A}$ is the velocity of particles in the decision subdomain A, w represents the inertia weight, $\vartheta_{1}$ and $\vartheta_{2}$ are the learning factors, and $\vartheta_{3}$ is the introduced repulsion factor, which affects the repulsive relationship between subdomains. $r_{1}$ and $r_{2}$ are random numbers between (0, 1), and $f_{Apb}$ is the current optimal value of particles in subdomain A. Applying repulsion between decision subdomains affects the interaction behavior of each subdomain, thereby improving the global optimization capability. natural()  is an exponentially decaying function, and the decay rate of the learning rate increases rapidly at first and then slows down as the number of iterations increases.

After the search in different subdomains is completed, the solution with a higher average fitness value and a more balanced optimal fitness value among decision subdomains is selected as the final solution for allocation. Assume that the mapping solution $f_{t}$ at time t satisfies a higher average fitness value and a more balanced optimal fitness value, and the calculation method for $f_{t}$ is shown in Equation (16).

(16) ft=argmaxt∈1…n ∑kfgbt+favgtfgbt−favgt  k∈1…q

where t is the timestamp during the search process, and k is the number of the decision subdomain.

### 4.3. Design of the SDN-MARA Algorithm

According to the four stages of the SDN-MARA algorithm based on the multi-domain controller game model proposed in this paper, four sub-algorithms are designed: fault controller detection, game domain construction, game-enhanced switch-to-target controller mapping, and out-of-control switch migration algorithm.

#### 4.3.1. Fault Controller Detection Algorithm

The paper adopts the adaptive fault detection protocol proposed by Fetzer et al. [24], which can be applied to the SDN control plane. The idea is to use the delay of heartbeat messages for fault judgment and analysis, thereby achieving controller fault detection. This detection process is dynamic and can be self-adjusted and optimized according to network conditions. This paper identifies suspected faulty controllers based on this protocol.

The process of detecting SDN controller faults based on this protocol is as follows: (1) In the SDN network, collect data related to the controllers, including controller log information, performance indicators (processing ability, capacity), network traffic, heartbeat information, etc. (2) Clean, filter, and organize the collected data. (3) Use a linear regression model for monitoring and judgment. During real-time monitoring, collect relevant data from the controllers and use the trained model to classify or predict the data. When an abnormality is detected, the fault detection mechanism is triggered, and corresponding measures are taken to handle the fault. The flowchart of the SDN controller fault detection algorithm is shown in Algorithm 1.
**Algorithm 1:** SDN Controller Fault Detection AlgorithmInput: Controller status information (INFOc), heartbeat packet delay information (INFOh)Output: Predicted fault status of the controller y_predInitialize linear regression modelmodel = Initialize();Define regression equation$y_{pred}=\theta_{0}+\theta_{1}*INFOc+\theta_{2}*INFOh;$Train model with training datasettrainingData = GetTrainingDataset()features = ExtractFeatures(trainingData)TrainModel(model, trainingData, features)get $\theta_{0},\theta_{1},\theta_{2}$$\theta_{0},\theta_{1},\theta_{2}$ = GetParameters(model)**For each** test sample t in TestDataset:testData = GetTestDataset()**For each** t in testData:y_pred = Predict(model, t)**If** |y_pred − t.trueLabel| > θ:Mark t as faulty**Return** y_pred

In the above table, INFOc signifies the status of the controller, INFOh denotes the heartbeat delay information, and *θ* is a scaling factor. The switches are under the direct control of the SDN controllers within their designated control domains. Upon detecting a faulty controller, the switches that it controls are promptly labeled as out-of-control.

#### 4.3.2. Game Domain Construction Algorithm

**Figure 3:**
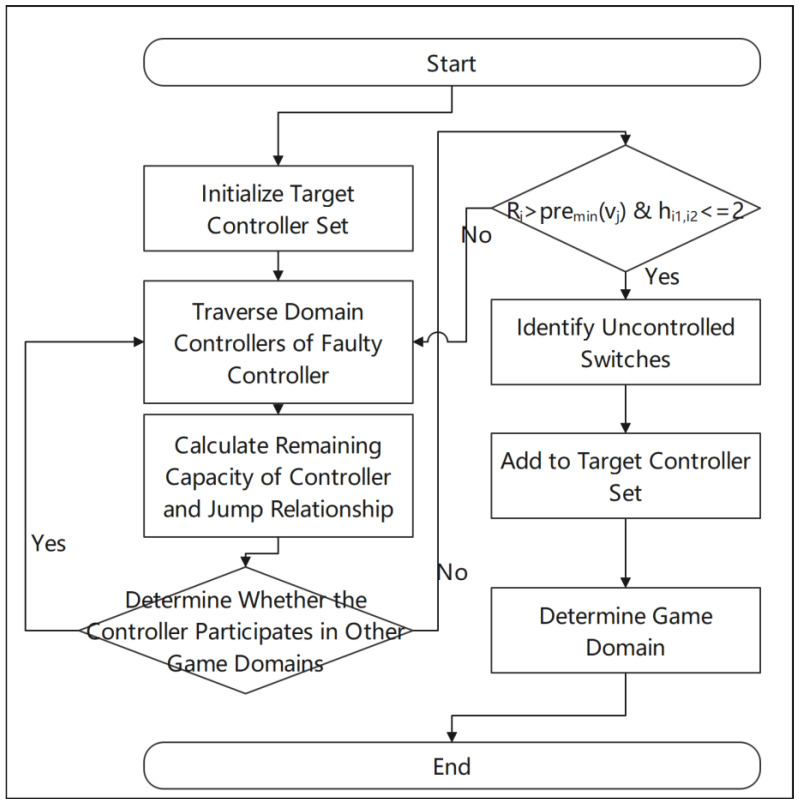
Flowchart for target controller selection and game domain construction.

The selection of the target controller is based on the remaining capacity $R_{i}$ of the controllers and the device’s transition relationships $h\left(i_{1},i_{2}\right)$. The steps for selecting the target controller and constructing the game domain are illustrated in Figure 3 below.

Firstly, initialize the set of target controllers by setting it to empty. Then, iterate through all the neighboring controllers of the faulty controller. Neighboring controllers are defined as those that meet $h\left(i,j\right)\leq 2$, that is, the transition relationship between the controller i and the switch j does not exceed 2. During the iteration, check whether the current controller is already involved in another game domain. If the controller is already involved, it will not be considered. For controllers that are not involved in any other game activities, calculate the remaining capacity $R_{i}$ of the relevant ones. The remaining capacity of the target controller needs to be greater than the minimum expected request rate ${pre}_{min}$ of the out-of-control switches, which is the condition that needs to be satisfied, as shown in Equation (17):

(17) $$R_{i}>{pre}_{min}\left(v_{i}\right)$$

Add all neighboring controllers that meet the conditions to the candidate set of target ones. Subsequently, add the out-of-control switches to this set. Finally, sort the controllers in the candidate set in descending order based on their remaining capacity. Assess the request demands of the out-of-control switches and, based on the assessment results, select one that meets the conditions from the candidate set as the final target controller set. The algorithm for constructing the game domain is presented in Algorithm 2.
**Algorithm 2:** Game domain construction algorithmInput: Controller status information INFOc, switch status information INFOv Output: Set of controllers in the game domain, Set of switches V0Initialize controllerSet to emptyFor each controller in neighboringControllers(faultyController):   If controller.isInvolvedInOtherActivities():   continue   If controller.remainingCapacity ≥ equation(14, switch.expectedRate) AND    distance(switch, controller) ≤ 2:   controllerSet.add(controller)For each switch in switches(faultyController):  If switch.isOutofControl():   gameDomain.addSwitch(switch)finalGameDomain = filterControllersBasedOnCapacity(controllerSet, switchInfo)

#### 4.3.3. Game-Enhanced Dynamic Mapping Algorithm for Switches and Target Controllers

Based on the SDN multi-domain controller game enhancement model proposed above, this paper designs a mapping algorithm to solve the control problem of out-of-control switches and promote their routing function. The algorithm is divided into four stages: (1) The out-of-control switch initiates a migration request to the target controller in the game domain; (2) The target controller comprehensively evaluates its remaining capacity and expected request processing capability, similar to routing decisions in traditional networks; (3) Perform the mapping selection process between switches and controllers to ensure the optimal routing path; (4) Establish the relationship between the switch and the target controller.

Inspired by the swarm intelligent optimization algorithm, this paper adopts the multi-population particle swarm optimization algorithm with repulsive interaction in game activities [7] to select the mapping between switches and controllers. The target controllers are abstracted as particles, and the set of target controllers in the game domain is further divided into several decision subdomains based on capacity and hop relationships. The mapping selection process between switches and controllers is divided into four stages, with different interaction factors applied between decision subdomains at different stages to achieve better results. The steps of the mapping selection algorithm are as follows:

1. Initialize the number of decision subdomains, the size of the particle swarm, and the particle positions x and velocities v. Each decision subdomain conducts independent searches, with particles within the subdomains calculating their fitness values based on Equation (10). The particles adjust their search velocities according to their individual best values and the global best value. During this stage, the search processes within each decision subdomain are completely isolated, thus exhibiting strong global optimization capabilities. Each decision subdomain obtains initial search results during this phase.
2. After obtaining the search results for each decision subdomain, intermittent repulsive forces are applied to different decision subdomains based on Definition 7 and Equations (14) and (15). These repulsive forces interfere with the interaction behaviors between subdomains, enhancing the global optimization capability.
3. In this stage, repulsive forces continue to be applied to the particles within the decision subdomains. Different from step 2, the repulsive forces between subdomains are applied in every iteration, causing the exploration behaviors of different subdomains to influence each other.
4. Following intense interactions and iterations among the decision subdomains, they begin to converge. The subdomains initially divided during the early stages of the algorithm are re-merged at this stage. The merged particle swarm integrates the previously searched results, and ultimately, the algorithm converges to obtain the optimal result. The global best value represents the optimal matching result between the migrating switch and the target controller.

The pseudocode for determining the mapping relationship between out-of-control switches and target controllers is shown in Algorithm 3.
**Algorithm 3:** GEDM Algorithm for Determining the Mapping Relationship between Out-of-Control Switches and Target ControllersInput: Target controller set $C_{t}$, out-of-control switch set $V_{o}$ Output: Mapping relationship $E_{1}$ between out-of-control switches and target controllersInitialize decision subdomain count $N_{d}$, particle position pos, and speed spGet the k = 1-th migrating switch and $A_{i}$ decision subdomainAbstract target controllers as particles for evolutionFor each particle in decision domains $A_{i}$ and $B_{i}$:**If** $f_{avg}\left(A_{i}\right)>f_{avg}\left(B_{i}\right)$ and $f_{gb}\left(A_{i}\right)>f_{gb}\left(B_{i}\right)$: **If** O$\left(A_{i}\right)>f_{pb}\left(A_{i}\right)$:  Update O$\left(A_{i}\right)$ **If** O$\left(A_{i}\right)>f_{gb}\left(A_{i}\right)$:  Update O$\left(A_{i}\right)$**Else if** $f_{avg}\left(A_{i}\right)<f_{avg}\left(B_{i}\right)$ and $f_{gb}\left(A_{i}\right)<f_{gb}\left(B_{i}\right)$: $f_{gb}\left(A_{i}\right)$ = $f_{gb}\left(B_{i}\right)$ $f_{pb}\left(A_{i}\right)$ = $f_{pb}\left(B_{i}\right)$**Else**: Update position and speed of particles in $A_{i}$ and $B_{i}$ using Equations (11) and (12)Establish mapping between the k-th switch and target controller Repeat Step 3 to Step 5 for all switches Update target controller set until all migrating switches are mapped Output mapping relationship $E_{1}$

#### 4.3.4. Out-of-Control Switch Migration Algorithm

After re-establishing the mapping relationship, fault recovery is carried out, which involves rebuilding control domains based on the new mapping relationship and setting up new communication channels between out-of-control switches and their corresponding target controllers. Once the above mapping relationship is established, a communication connection needs to be set up, and the out-of-control switch must be registered into the local control domain to complete the migration process. The out-of-control switch migration algorithm is shown in Algorithm 4.
**Algorithm 4:** Out-of-control Switch Migration Algorithm**Input:** Controller and switch mapping relationship $E_{1}$ **Output:** Switch migration resultsController actively sends HELLO message to negotiate version and other informationController sends Features message to retrieve basic status information of faulty switchesController obtains the topology of the region where the faulty switch is located and installs flow tablesFaulty switch updates flow tables and saves network topology information Controller updates the list of switches within the domain and switches the faulty switch to the local controller

After establishing a communication connection between the migrating switch and the target controller, it signifies that the switch has completed its migration task. The target controller then broadcasts an update message to the entire control plane. Once all out-of-control switches have completed their migration tasks, the game domain is automatically dissolved.

## 5. Experiments

### 5.1. Experimental Design and Environment

The SDN network simulation experiment is conducted on the Ubuntu 24.04 operating system. The Floodlight [25] controller serves as the control platform, while Mininet [26] is used to generate experimental network topologies. Iperf [27] is utilized for assessing network performance metrics, and the cbench [28] tool is employed to test the load pressure variations on the target controller. Additionally, NetworkX [29] is used to construct network topologies to validate the effectiveness of the proposed algorithm.

To evaluate the performance of the proposed algorithm, various network topologies from the literature [30] with differences in the number of switches and links reflecting the diversity of the experimental setups are adopted. The network topology information for the experiments is detailed in Table 1. The capacity limit of the controllers in the experimental environment is set to handle a maximum of 8000 packet_in messages, while the request rate from switches is maintained between 200 and 400 messages per second. To ensure the scientific accuracy and correctness of the experimental results, a total of 200 experiments were conducted under different network scales, and finally, average values of the experimental data were measured as verification results. In this experiment, a timeout-based retransmission strategy was implemented to prevent packet loss. After a controller sends a route update to a neighboring controller, it awaits confirmation. If no confirmation is received within five seconds, the update is retransmitted.

The proposed algorithm in this paper is compared with the CFRS [31], BCS [30], and SBC [32]. The core idea of CFRS is to execute a backup controller adjustment algorithm based on a multi-population particle swarm, which reduces the average propagation delay between out-of-control switches and backup controllers. The BCS algorithm, based on a heuristic approach, selects a new target controller for out-of-control switches while minimizing the activation overhead of controllers under the premise of load balancing, thereby enhancing recovery efficiency. SBC employs a shared data method to store network states during failures, calculates the number of backup controllers based on probability theory, and uses a multi-objective optimization algorithm to determine the placement scheme for backup controllers.

In contrast, this paper comprehensively considers the remaining capacity of the target controller and the migration cost of out-of-control switches, allowing for the dynamic selection of the optimal mapping between them while ensuring load balancing among the target controllers. The effectiveness of the proposed algorithm is validated from three aspects: the migration cost of switches, the load situation of target controllers, and the convergence performance of the algorithm.

### 5.2. Experimental Result Analysis

#### 5.2.1. Comparison of Migration Costs for Out-of-Control Switches

**Figure 4:**
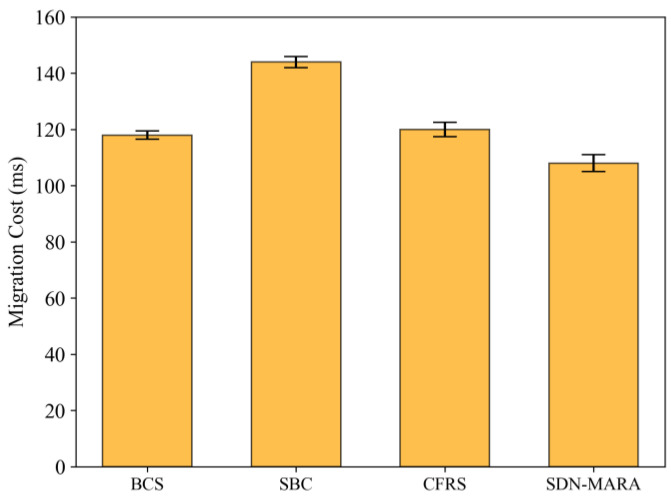
Comparison of migration costs for out-of-control switches.

Figure 4 illustrates the comparison of migration costs for out-of-control switches across different algorithms. From the figure, it is evident that the SBC algorithm incurs the highest migration cost. This is primarily due to its shared mechanism for fault recovery, which increases the communication overhead between out-of-control switches and target controllers during migration operations across different network domains. The performance of BCS in out-of-control switch migration cost is basically the same as that of CFRS and slightly better than that of SBC. This is because the former relies on a minimal set of target controllers while also considering the reduction of their activation costs, thus lowering communication overhead. In contrast, the CFRS algorithm takes into account both controller load and propagation delay when selecting target controllers. The proposed SDN-MARA algorithm demonstrates a lower overall migration cost for out-of-control switches compared to the other three. This is attributed to its comprehensive consideration of the costs of establishing secure channels and migration rules during the mapping selection phase between out-of-control switches and target controllers.

#### 5.2.2. Comparison of Fault Recovery Experiments

**Figure 5:**
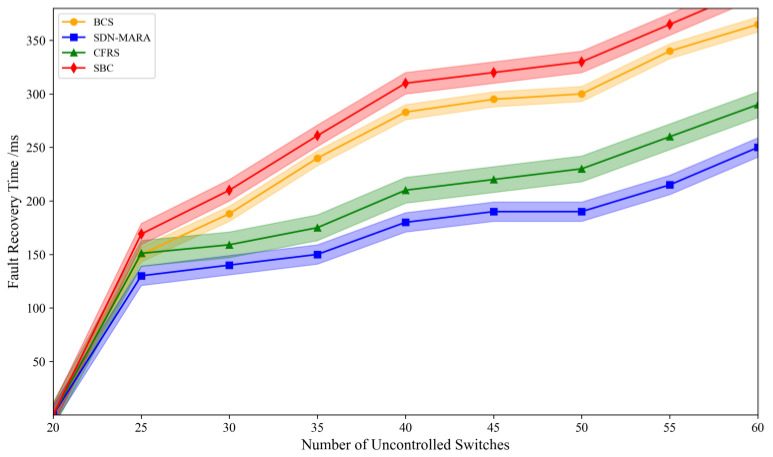
Comparison of fault recovery delays.

Figure 5 presents a comparison of fault recovery delays across different algorithms. It is evident from the figure that an increase in the number of faulty controllers leads to a corresponding rise in the number of out-of-control switches, which in turn results in increased fault recovery delays. Both the BCS and SBC algorithms are based on backup controller selection strategies and seek to identify a set of target controllers under load constraints. However, due to BCS’s approach of minimizing the activation overhead of controllers, the overall fault recovery delay for the SDC algorithm is greater than that of the BCS algorithm.

The CFRS algorithm significantly reduces the fault recovery delay compared to the BCS and SBC. As illustrated in the figure, CFRS exhibits comparable fault recovery delays to the proposed SDN-MARA algorithm when the scale of out-of-control switches is small. However, as this number increases, the SDN-MARA algorithm demonstrates a marked advantage. Specifically, it can reduce the delay by up to 11% and averages a reduction of 6.5%. This improvement is attributed to the continuous optimization of target controller selection facilitated by the multi-population particle swarm algorithm with repulsive interaction. Particularly, the interaction mechanism of multiple populations in phases two and three enhances the algorithm’s ability to identify global optimal efficiently, thereby saving optimization time. Additionally, the SDN-MARA algorithm takes into account both the migration costs of out-of-control switches and the load pressures on target controllers, resulting in shorter fault recovery delays compared to the other three algorithms.

#### 5.2.3. Comparison of Target Controller Load Pressure

**Figure 6:**
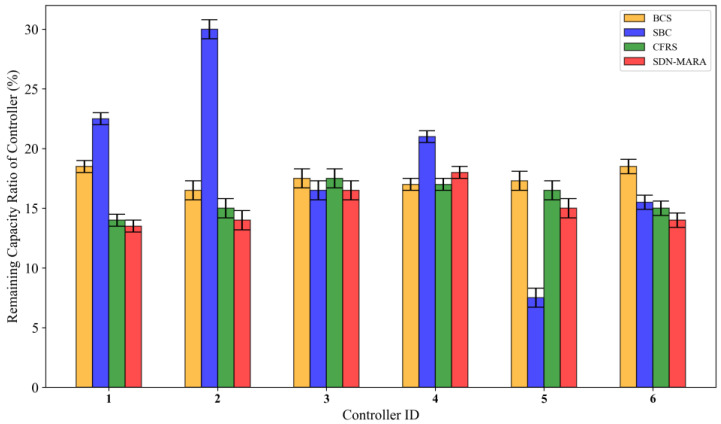
Remaining capacity ratio of target controllers.

Figure 6 presents a comparative experiment on the remaining capacity ratio of target controllers after taking over the out-of-control switches. As shown in the figure, the remaining capacity ratio of target controllers under the SDN-MARA algorithm remains relatively consistent. This is primarily because the algorithm considers the remaining capacity of all controllers participating in the decision-making process when selecting target controllers for out-of-control switches. Controllers with larger remaining capacity will take over more out-of-control switches, ultimately achieving a balanced distribution of the load among target controllers.

The BCS algorithm fails to update the remaining capacity of target controllers in time, resulting in an overall load performance that is lower than that of the SDN-MARA algorithm. The SBC algorithm does not account for the load of target controllers, leading to a highly uneven distribution of load among them. The performance of the CFRS algorithm is generally consistent with that of the SDN-MARA algorithm, as CFRS achieves a uniform distribution of out-of-control switches by setting load ranges for target controllers and continuously updating their load.

## 6. Conclusions

This paper addresses the reliability issues of controllers in an SDN multi-domain controller environment by proposing an SDN-MARA algorithm. Comparative experimental results demonstrate that the proposed model and algorithm show advantages in terms of the migration cost of out-of-control switches, fault recovery delay, and remaining capacity of target controllers. Future research will focus on the consistency of data across control nodes when multiple controller fails, controller migration, and domain restructuring, as well as extending the autonomous failure recovery algorithm for controllers to distributed computing.

## Figures and Tables

**Table 1:** Network topology information.

Topology Name	Number of Switches	Number of Links	Number of Controllers
NT1	30	31	6
NT2	50	89	6
NT3	70	121	6
NT4	90	152	6
NT5	120	239	6

## Data Availability

Data are contained within the article.
